# Computer classification and construction of a novel prognostic signature based on moonlighting genes in prostate cancer

**DOI:** 10.3389/fonc.2022.982267

**Published:** 2022-10-07

**Authors:** Wangli Mei, Liang Jin, Bihui Zhang, Xianchao Sun, Guosheng Yang, Sheng Li, Lin Ye

**Affiliations:** ^1^ Department of Urology, Shanghai East Hospital, School of Medicine, Tongji University, Shanghai, China; ^2^ Department of Urology, Shanghai Tenth People’s Hospital, School of Medicine, Tongji University, Shanghai, China; ^3^ Department of Biochemistry, Dalian Medical University, Dalian, China

**Keywords:** moonlighting genes, prostate cancer, prognostic signature, immune microenvironment, biochemical failure-free survival

## Abstract

Advanced prostate cancer (PRAD) patients have poor prognosis and rising morbidity despite the ongoing iteration of molecular therapeutic agents. As newly discovered proteins with several functions, Moonlighting proteins have showed an important role in tumor progression but has not been extensively investigated in PRAD. Our study aimed to identify moonlighting-related prognostic biomarkers and prospective PRAD therapy targets. 103 moonlighting genes were gathered from previous literatures. A PRAD classification and multivariate Cox prognostic signature were constructed using dataset from The Cancer Genome Atlas (TCGA). Subsequently, we tested our signature’s potential to predict biochemical failure-free survival (BFFS) using GSE21032, a prostate cancer dataset from Gene Expression Omnibus (GEO). The performance of this signature was demonstrated by Kaplan-Meier (KM), receiver operator characteristic (ROC), areas under ROC curve (AUC), and calibration curves. Additionally, immune infiltration investigation was conducted to determine the impact of these genes on immune system. This signature’s influence on drug susceptibility was examined using CellMiner’s drug database. Both training and validation cohorts demonstrated well predictive capacity of this 9-gene signature for PRAD. The 3-year AUCs for TCGA-PRAD and GSE21032 were 0.802 and 0.60 respectively. It can effectively classify patients into various biochemical recurrence risk groups. These genes were also assessed to be connected with tumor mutation burden (TMB), immune infiltration and therapy. This work created and validated a moonlighting gene signature, revealing fresh perspectives on moonlighting proteins in predicting prognosis and improving treatment of PRAD.

## Introduction

Generally, the prevalence of PRAD has been growing in recent decades. According to a recent survey, prostate cancer has risen to become the second most frequent disease in males globally and the fifth top reason of tumor-related mortality in men (1). PRAD was diagnosed in the early stages in approximately 93% of the cases, with 5-year survival rate approach to 100% (2). In contrast, the five-year survival rate for individuals with advanced tumors was only around 30% (3). Advanced PRAD included Gleason score 8-9, pathological T stage T3-4, pathological N stage N >= 1, as well as M stage M >= 1 (4). The Prostate-specific antigen (PSA) test was applied for the early diagnosis of PRAD in early 1990s, and the number of cases of prostate cancer diagnosed substantially rose. When it comes to screening for PRAD, PSA is now best first-step serum test, since it remains the most often utilized biomarker (5). Although PSA has achieved outstanding achievements in early diagnosis of PRAD, there is no unanimity as to if PSA may lower risk of mortality in PRAD patients (6). Thus, it is crucial to identify biomarkers for early detection of PRAD and as prospective therapeutic targets for reducing the aggressiveness of PRAD, preventing distant metastasis, and improving patient outcomes. Additionally, molecular imaging, such as multiparametric magnetic resonance of the prostate (mpRNA, a radiologic technique), has played an important role in the diagnosis, stage, Gleason score and treatment of PRAD (7, 8). Although radical prostatectomy (RP) has been regarded as the most effective therapy for PRAD, around 30% of individuals still develop to biochemical recurrence (BCR) following RP (9). Having a current PSA concentration > 0.2ug/L after RP is classified as having a BCR, and it may be a sign of metastasis as well as a poor prognostic predictor (10). It is forecasted that around 40% of BCR patients following RP will die from PRAD within 15 years (11). So, early prediction of BFFS for PRAD patients was crucial for selecting the optimum therapeutic method and preventing PRAD development. Currently, standard radiation and chemotherapy were used to treat PRAD, particularly serious PRAD (12). Nevertheless, for people with serious PRAD, the overall effectiveness was remained inadequate. As a result, accurate new diagnostic markers and prognosis models are urgently required to increase the credibility of early diagnosis and targeted therapy. The term moonlighting proteins refers to proteins with several functions. There are over a hundred proteins, including as enzymes, receptors, saffolds, transcription factors, etc., that have a secondary function. The moonlighting proteins have a wide range of biological roles, including control of transcription, apoptosis, DNA splicing, and DNA repair. Yet, the mechanism by which moonlighting genes contribute to tumor growth remained disputed and unclear (13). It is still unknown if the moonlighting gene signature is substantially relevant with the prognosis of PRAD patients and their immune microenvironment or treatment.

It was the goal of our study to develop a prognostic signature in PRAD patients based on moonlighting genes in ability to forecast the BFFS of PRAD patients, as well as to determine whether the signature could regulate the immune microenvironment and serve as a potential therapeutic to improve prognosis of PRAD patients.

## Materials and methods

### Data acquisition

MoonProt database was used to obtain the 103 moonlighting genes for Homo sapiens, which are listed in **Supplementary Table 1** (14). RNA sequencing (RNA-seq) and clinical data were acquired from TCGA (15) and GEO (GSE21032). The TCGA cohort contained 551 PRAD samples (52 normal samples and 499 tumor samples), while the GEO cohort, which served as an external validation, contained 185 tumor samples. All samples were collected after RP with detailed clinical data and follow-up information. Received RNA-seq data consisted of row-counts. Clinical information covered age, tumor stage, Gleason score, and other variables. We identified moonlighting-related differentially expressed genes (mDEGs) that were statistically significant with p value< 0.05 (16). Immune, estimate, and stromal score of TCGA-PRAD samples (**Supplementary Table 2**) were obtained from Estimation of Stromal and Immune cells in Malignant Tumor tissues using Expression (ESTIMATE) (17). 79 immunological checkpoint genes (ICGs, **Supplementary Table 3**) were obtained from published literature (14, 18, 19).

### Construction of a signature for predicting prognostic of individuals with PRAD

The BFFS time of mDEGs in TCGA with p< 0.05 was evaluated using univariate Cox regression analysis, and 16 prognostic-related mDEGs were discovered as candidate genes. To narrow the scope of candidate genes, the R package “glmnet” was applied in conjunction with penalty parameter (λ) to perform least absolute shrinkage and selection operator (LASSO) Cox regression analysis (20). These genes were utilized to explore connections between gene expression and PRAD subtypes. KM curves were drawn to illustrate the BCR difference between different clusters. Additionally, to evaluate the clinical features and gene expression levels in different clusters, we constructed a heatmap.

Each target gene’s hazard ratio (HR) and 95 percent confidence interval (CI) were calculated using “survminer” package. The genes’ regression coefficients were obtained by multivariate Cox regression analysis, and risk score was calculated using following equation: $risk\;score\;=\;\sum_{i}^{\text{n}}\;Xi\;*\;Yi\;$ (*X*: coefficients, *Y*: gene expression). The median risk score was applied to split patients into 2 clusters with low and high-risk. In addition, principal component analysis (PCA) was utilized to see whether these patients were properly separated into two clusters. The predictive efficacy of this signature in predicting the BFFS of PRAD patients was evaluated by KM survival curves and time-dependent ROC.

### Internal and external validation of this signature and clinical correlation analysis

The clinical information of age, tumor stage, and Gleason score were all taken from the TCGA cohort for internal confirmation. Additionally, the GEO cohort provided the clinical data for external validation (GSE21032). In order to determine whether or not this signature could be considered the independent prognostic factor, both univariate and multivariable Cox regression analyses were performed on the data. After multivariate Cox regression analysis (21), a nomogram was produced using the outcomes and a calibration curve was generated to verify this nomogram’s accuracy.

Correlations between PRAD clinical features and risk scores, as well as gene expression levels from this predictive signature, were evaluated using Pearson’s correlation analysis. Furthermore, KM curve was employed based on this signature to measure the BFFS of PRAD patients in different stages.

### Gene set enrichment analysis

It was decided to use gene set enrichment analysis (GSEA) to analyze relative enrichment of certain gene sets across a sample population and to detect the statistically differential expression patterns between different risk groups in our study (22).GSEA software (version 4.1.0) was obtained from https://www.gsea-msigdb.org/gsea/msigdb/index.jsp (23). The GSEA was carried out with the help of Molecular Signatures Database (MSigDB) 7.5.1 version. In the categories of Biological Process (BP), Cellular Component (CC), Molecular Function (MF), Kyoto Encyclopedia of Genes and Genomes (KEGG) pathways, and immunologic signature gene sets, the gene sets enrichment analysis was conducted. Gene sets having a normal of P-value< 0.05 were regarded to be statistically differential gene sets in this study.

### Relationship between this signature and tumor mutation burden (TMB)

TCGA’s single nucleotide variation (SNV) dataset of PRAD patients in the form of masked somatic mutations was then acquired to investigate correlation between TMB of PRAD patients and risk score derived from this signature (Workflow Type: aliquot ensemble somatic variant merging and masking) (24, 25). “Maftools” R package was employed to analyse TMB scores of individuals with PRAD, and the association between subgroups was assessed.

### Evaluation of tumor immune microenvironment

An R package called “limma” was utilized to obtain genes that were expressed differently in individuals with high and low levels of risk considering |log2FC| ≥ 1 and FDR< 0.05. Gene Set Variation Analysis (GSVA) (26) was employed to analyze immune cells infiltrating and immune pathways by “GSVA” R package with single-sample gene set enrichment analysis (ssGSEA, https://software.broadinstitute.org/cancer/software/genepattern/). Additional to this, univariate Cox regression analysis was conducted on these DEGs in order to find those that were prognostic-related and scored higher with p< 0.001. The “corr.test” function in the “psych” R package was utilized to examine correlation between these genes and immune cells or immune-related pathways. Immunological-related scores and 79 ICGs were subjected to a correlation analysis in order to learn more about the link between the signature and the immune microenvironment with p< 0.05.

### Drug sensitivity analysis

Using the CellMiner database, which contains 60 different cancer cell lines from nine different malignancies, we were able to obtain drug sensitivity information for the National Cancer Institute (NCI) 60 in United States (27). The drugs that had been approved by Food and Drug Administration or were currently in clinical development were chosen for further investigation. Under the help of Pearson’s correlation analysis, we were able to assess relationship between this signature and drug sensitivity.

### Statistical analyses

PRAD patients in TCGA and GEO cohorts were investigated using univariate and multivariate Cox regression analysis to construct one signature that predicted prognosis. Patients’ prognosis was assessed using their BFFS. The gene signature’s prognostic value was demonstrated using the KM curve, ROC curve and Cox regression analysis. Chi-square tests, corrected chi-square tests, and Pearson’s correlation analysis were employed to compare continuous data between two groups. P< 0.05 was used to determine statistical significance for all of our analyses. All statistical analyses were carried out by R 4.41 (packages: limma, glmnet, survminer, prcomp, timeROC, rsm, Maftools, psych, etc.).

## Results

### Identification of prognostic moonlighting-related genes in TCGA-PRAD

**Figure 1:**
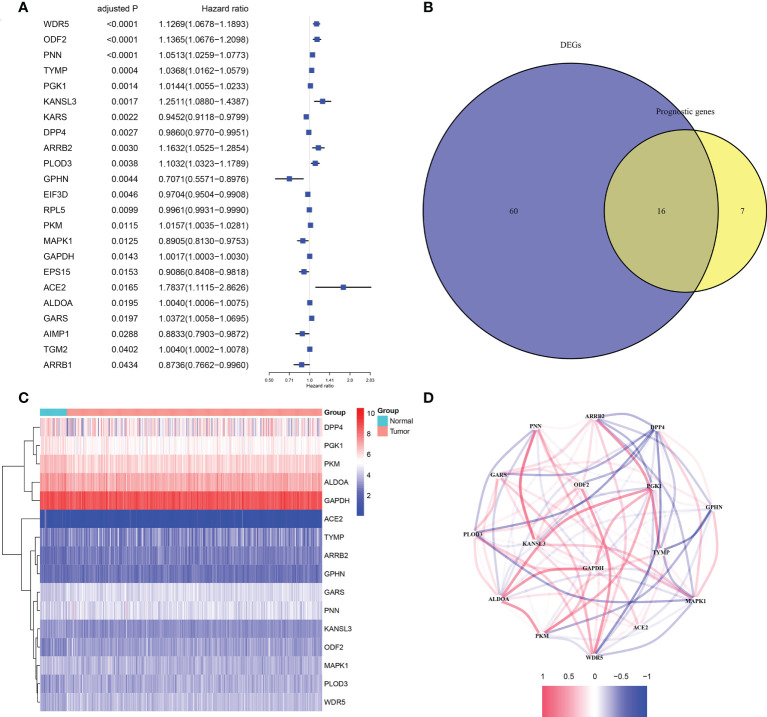
The candidate moonlighting genes in TCGA. **(A)** Univariate Cox regression analysis of PRAD for 23 prognostic moonlighting genes with P< 0.05. **(B)** Identification of mDEGs correlated with BFFS. **(C)** The expression of 16 prognosis-related mDEGs between normal and tumor samples. **(D)** The correlation network of 16 candidate genes.

According to the results of differential analysis in TCGA cohort, 76 moonlighting-related genes (**Supplementary Table 4**) were recognized as being differently expressed between normal and prostate cancer tissues with p< 0.05 and FDR< 0.05. Also, univariate Cox regression inferred 23 predictive genes with the same criterion (**Figure 1A**). A heatmap revealed expression levels of 16 overlapping genes in paracancerous and cancerous prostate tissues (**Figures 1B, C** **)**. A correlation network of 16 genes was established to examine relations between them (**Figure 1D**).

### Tumor classification

**Figure 2:**
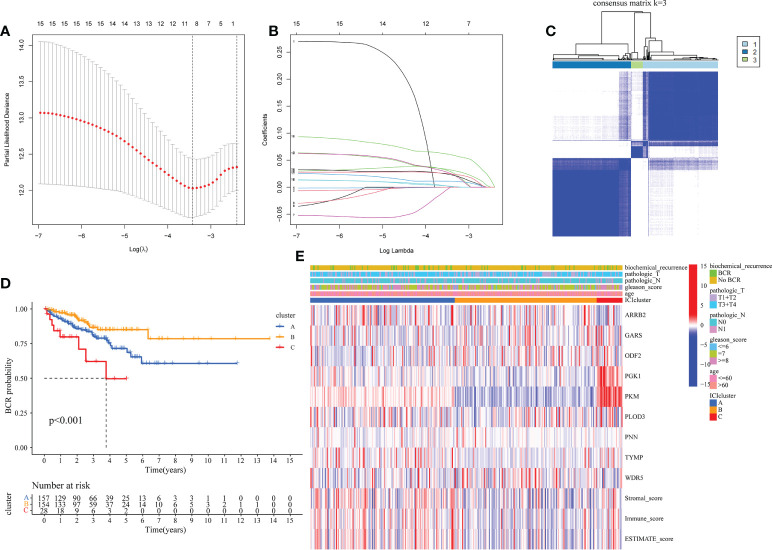
Classification of PRAD based on moonlighting genes in TCGA. **(A, B)** Lasso regression analysis of 16 candidate moonlighting genes, and 9 genes were excluded for the signature. **(C)** The classification of PRAD patients based on the consensus clustering matrix (k = 3). **(D)** KM curves of BFFS between 3 clusters. **(E)** Heatmap of the clinicopathologic features and 9 genes in 3clusters.

Due to the outcomes of the univariate Cox regression and differential analyses, 16 mDEGs were revealed to be linked with prognosis of PRAD patients. Next, the LASSO regression analysis was performed to exclude genes for consideration in order to establish a favorable signature (**Figures 2A, B** **)**, and a 9-gene signature was generated. For the purpose of investigating relationships between expression of these genes and the different PRAD groups, we conducted a consensus clustering analysis with PRAD patients from TCGA. Through a series of iterations of raising the clustering variable (k) from 2 to 10, we discovered that when k = 3, these patients could be effectively classified into 3 groups based on these genes (**Figure 2C**). 3 clusters were also analyzed for the BFFS time and clearly distinct discrepancies were observed (P< 0.001, **Figure 2D**). Gene expression levels, clusters, clinical characteristics, including age (≤60 or >60 years), Gleason score (<=6, = 7 or >=8), pathological T/N stage (T1+T2 or T3+T4, N0 or N1), and BCR status (BCR or No BCR) were shown in a heatmap, and some differences in clinical features across three clusters were discovered (**Figure 2E**).

### Construction and validation of moonlighting-related gene signature in TCGA

**Figure 3:**
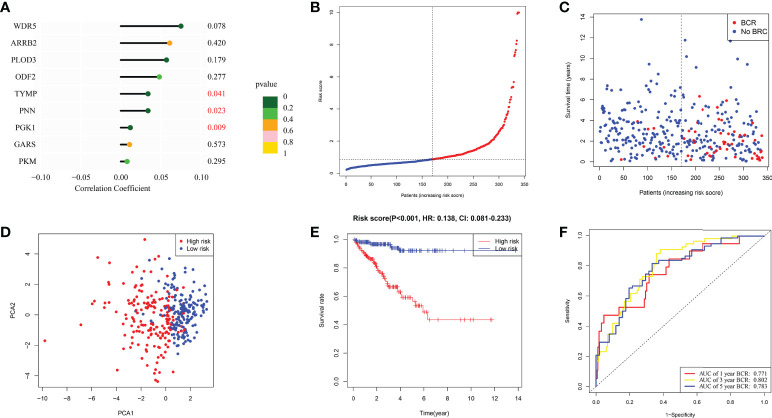
The construction of risk signature in TCGA. **(A)** Coefficient of 9 genes in this signature. **(B, C)** Distribution, BFFS status and risk score of PRAD patients. **(D)** PCA graph for PRAD patients. **(E)** KM curve for BFFS of PRAD patients based on high and low-risk groups. **(F)** ROC curves of 1, 3, 5-year for this signature.

Following is formula for calculating risk score associated with this signature using multivariate Cox regression analysis: risk score = (0.061* *ARRB2* exp.) + (-0.010* *GARS* exp.) + (0.048 * *OFD2* exp.) + (0.012* *PGK1* exp.) + (0.008* *PKM* exp.) + (0.057* *PLOD3* exp.) + (0.034 * *PNN* exp.) + (0.034* *TYMP* exp.) + (0.075* *WDR5* exp.) (**Figure 3A**). The 339 individuals (those with missing clinical data and BCR data excluded) were classified into 170 low and 169 high-risk categories. BCR status plot revealed that high-risk individuals had more BCR samples and shorter BCR time (**Figures 3B, C** **)**. Moreover, PCA revealed that these individuals were effectively separated into two groups (**Figure 3D**). In accordance with such a finding from KM curve, we discovered that this signature was substantially associated with BFFS of PRAD patients, with BFFS in high-risk group being much shorter (**Figure 3E**, p< 0.001, HR: 0.0138, CI: 0.081- 0.233). Besides, AUC values of 1-, 3-, and 5-years BFFS assessed by this signature were around 0.771, 0.802, and 0.771, respectively (**Figure 3F**).

**Figure 4:**
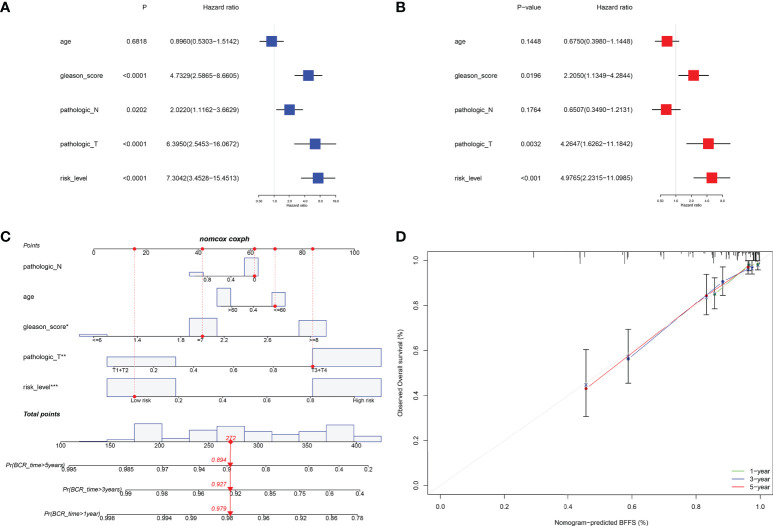
Internal validation of risk signature in TCGA. **(A, B)** Univariate and multivariate Cox regression analysis in TCGA cohort. **(C)** Nomogram based on this signature for 1, 3, 5-year BFFS prediction (The red point represented the prediction of one patient in TCGA cohort). **(D)** Calibration graph for agreement test between 1, 3, 5-year BFFS prediction and actual observation.

Internal validation was performed using TCGA data. **Table 1** displayed the clinical features of these individuals and the outcomes of Pearson’s correlation analysis. This signature was shown to be substantially linked with the development of PRAD, specifically in pathologic T/N stage, Gleason score, and BCR status. The univariate and multivariate Cox regression analyses were used to assess if our signature could be one independent prognostic factor to predict BFFS in PRAD patients. Following a univariate Cox regression analysis, we discovered that Gleason score, pathologic T/N stage, and risk score (p< 0.001, HR = 7.304, 95% CI: 3.453-15.451) were all potentially predictive markers for PRAD patients in TCGA (**Figure 4A**). After performing a multivariate Cox regression analysis, it is possible that this risk score associated with this signature was identified as the independent prognostic factor (p< 0.001, HR = 4.977, 95% CI: 2.232-11.099, **Figure 4B**). Prognostic nomogram analysis revealed that this signature had excellent prediction performance for BFFS of PRAD (**Figure 4C**). In order to comprehend the accuracy of such a signature, the calibration curve was drawn to represent the likelihood of BCR at 1, 3, or 5 years in PRAD patients, and the outcome demonstrated the optimum agreement between the forecast of the nomogram and the actual observation (**Figure 4D**).

**Table 1:** Clinical characteristics of PRAD patients in different risk clusters.

Clinical characteristics (samples)		TCGA cohort (339)			GSE21032 cohort (115)		
		Low risk	High risk	p value	Low risk	High risk	P value
Age (years)	<=60	78 (23%)	69 (20%)	0.407	38 (33%)	34 (30%)	0.647
	>60	92 (27%)	100 (29%)		20 (17%)	23 (20%)	
Pathologic N	N0	159 (47%)	122 (36%)	<0.001	55 (48%)	48 (42%)	0.074
	N1	11 (3%)	47 (14%)		3 (3%)	9 (8%)	
Pathologic T	T1+T2	78 (23%)	39 (12%)	<0.001	38 (33%)	30 (26%)	0.224
	T3+T4	92 (27%)	130 (38%)		20 (17%)	27 (23%)	
Gleason score	<=6	14 (4%)	5 (1%)	<0.001	15 (13%)	13 (11%)	0.025
	=7	116 (34%)	54 (16%)		38 (33%)	28 (24%)	
	>=8	40 (12%)	110 (32%)		5 (4%)	16 (14%)	
BCR	No	162 (48%)	121 (36%)	<0.001	49 (43%)	33 (29%)	0.003
	Yes	8 (2%)	48 (14%)		9 (8%)	24 (21%)	

P value was calculated by Pearson’s correlation to evaluated correlation between signature and clinical characteristics.

### External validation of this risk prognostic signature in GEO

**Figure 5:**
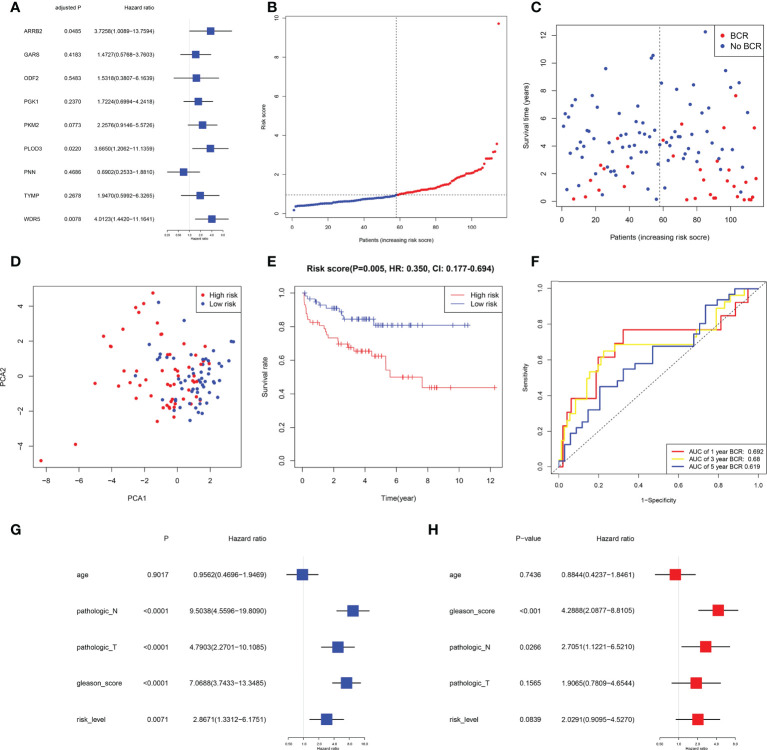
External validation of risk signature in GEO cohort. **(A)** Univariable Cox regression of 9 genes in GEO cohort. **(B, C)** Distribution, BFFS status and risk score of PRAD patients. **D** PCA graph for PRAD patients. **(E)** KM curve for BFFS of PRAD patients based on subgroups. **(F)** AUC of time-dependent ROC curves. **(G, H)** Univariate and multivariate Cox regression analysis in GEO cohort.

Data for samples used for external validation were obtained from GEO (GES21032), which included 179 PRAD samples in total. After excluding the individuals who did not have full clinical or BFFS data, 115 patients were chosen (**Table 1**). In the GEO cohort, the risk score was shown to be substantially linked with the Gleason score and the BCR based on correlation analysis. The prognostic values of these genes in GEO were assessed by univariate Cox regression analysis (**Figure 5A**). On the basis of identical technique described above, these individuals were likewise classified into 58 low and 57 high-risk categories. Meanwhile, patients in high-risk cluster were more prone to BCR and had shorter BFFS time (**Figures 5B, C** **)**. According to the results of the PCA, we could find that these patients were also well split into two clusters of patients (**Figure 5D**). The KM curve revealed a substantial difference between two groups’ BFFS (**Figure 5E**). ROC curve analysis in this cohort illustrated that our model had significant prognostic value with AUC = 0.692 for 1-year, 0.680 for 3-year, and 0.619 for 5-year BCR (**Figure 5F**). This signature might potentially serve as a useful predictor of BFFS in patients with PRAD, as shown by results of univariate and multivariate Cox regression analysis (**Figures 5G, H** **)**. Overall, this signature could well predict postoperative BCR in patients with PRAD.

### Clinical correlation of the signature in PRAD patient

**Figure 6:**
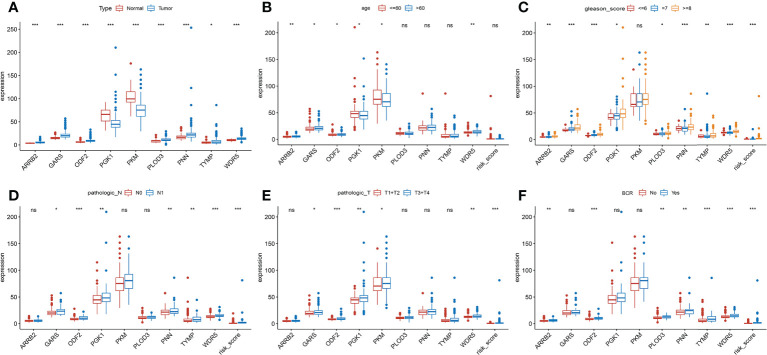
Clinical association analysis of this signature in TCGA. **(A)** Differential expression of 9 genes between normal and tumor tissues. **(B-F)** The association between the signature and clinical characteristics. (ns, p >= 0.05; *0.01 <= p < 0.05; **0.001 <= p < 0.01; ***p <0.001).

**Figure 7:**
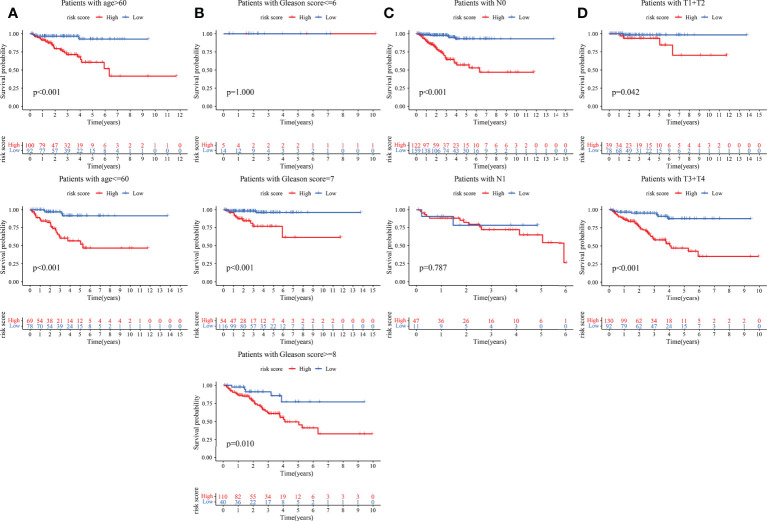
KM curves based on clinical features for BFFS of this signature in TCGA. The KM curves of patients based on ages **(A)**; Gleason scores **(B)**; pathological stage **(C)** and pathological stage **(D)**.

The clinical features were correlated with our signature genes (*ARRB2*, *GARS*, *ODF2*, *PGK1*, *PKM*, *PLOD3*, *PNN*, *TYMP*, *WDR5*) and risk score according to Pearson’s correlation analysis. The result of the differential analysis was shown in **Figure 6A**, *ARRB2*, *GARS*, *ODF2*, *PLOD3*, *PNN*, *TYMP* and *WDR5* were upregulated in tumor tissues, while *PGK1* and *PKM* were downregulated. Furthermore, expression levels of majority of genes were shown to be substantially linked with the clinical features of PRAD. Particularly, the risk score grew in conjunction with the development of the tumor in this study (p value of Gleason score, pathologic T staging, pathologic N staging and BCR all< 0.001, **Figures 6B–F**). Then, to further investigate the predictive usefulness of this signature in PRAD patients with varying clinical features, we used KM curve analysis in patients at various stages. According to the results of the analysis, we could find that the BFFS time of patients with different age, Gleason score >=7, pathologic N0 stage and different pathological T stage had significant BFFS difference in different risk clusters (**Figure 7**). The outcomes of patients with N1 and Gleason score = 6 were unfavorable, perhaps because to the limited sample size. In general, this signature provided a decent prediction impact for the majority of patients with different stages of PRAD.

### Possible biological mechanisms of the signature in PRAD

**Figure 8:**
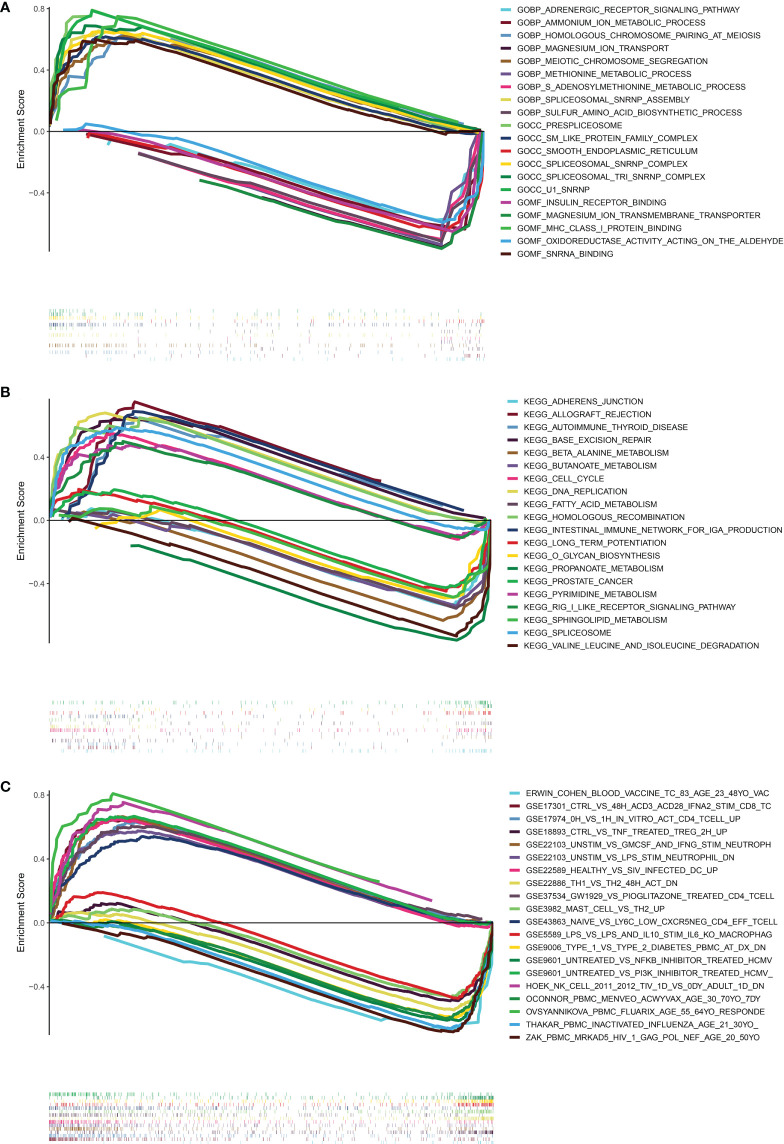
Results of GESA in TCGA. **(A)** The results of GSEA in GO (BP, CC, MF). **(B)** The results of GSEA in KEGG. **(C)** The results of GSEA in immunologic signature gene sets. (curves above horizontal line: positive correlation; the curve below horizontal line: negative correlation; the heatmap below curves: the enrichment degree of these pathways or gene sets in PRAD patients).

In order to investigate the possible pathways that were substantially associated with this signature in PRAD, GSEA was applied between high and low-risk TCGA clusters. When a nominal p value< 0.05 was found, gene sets were regarded differentially enriched. GSEA revealed significant differences in enrichment of MSigDB collection (c5.go.v7.5.symbols.gmt, c2.cp.kegg.v7.5.symbols.gmt, c7.immunesigdb.v7.5.symbols.gmt). Results of GSEA for Gene Ontology (GO), KEGG and immunesigdb were shown in **Figures 8A–C** respectively. We found that GOCC U1 SNRNP (normalized enrichment score, NES = 2.19), GOBP HOMOLOGOUS CHROMOSOME PAIRING AT MEIOSIS (NES = 2.14), KEGG CELL CYCLE (NES = 1.78) and KEGG BASE EXCISION REPAIR (NES = 1.91) were more active in high-risk group.

### The relationship between risk score and TMB

**Figure 9:**
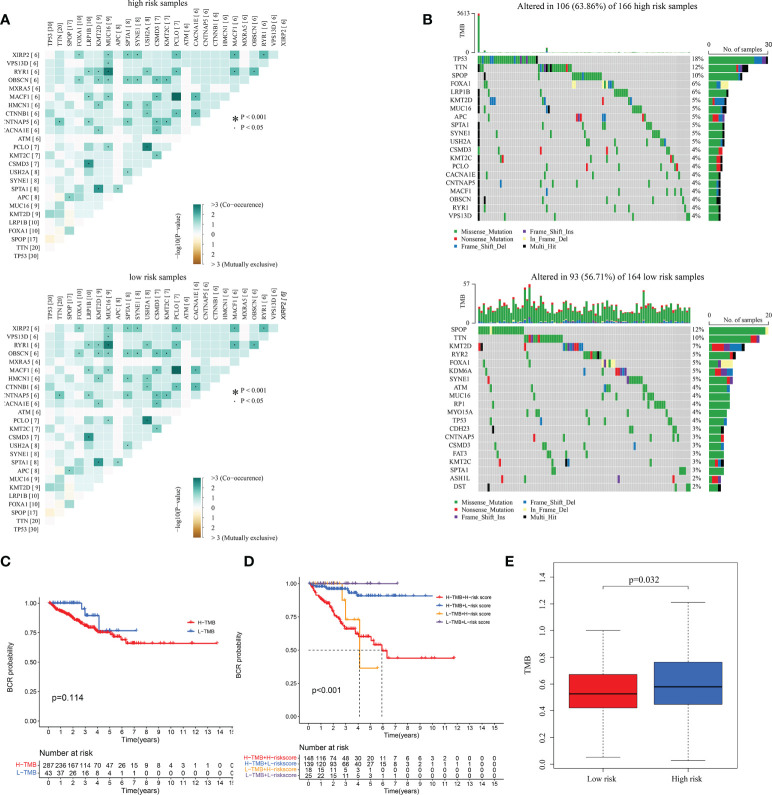
The correlation between TMB and the signature in TCGA. **(A)** The heatmaps of the correlation between some common mutated genes in both high-risk and low-risk groups. **(B)** Waterfall plots mutated genes in both subgroups. **(C, D)** KM curves of patients with different TMB and risk levels. **(E)** The differential analysis of TMB between different risk groups.

To understand correlation between some common mutated genes with different risk levels, the heatmaps were drawn in high and low-risk clusters (**Figure 9A**). Waterfall maps were constructed to evaluate genetic mutation difference between subgroups, and results demonstrated that *TP53*, *TNN* and *SPOP* had higher mutation rate in high-risk groups, while *SPOP*, *TTN* and *KMT2D* had higher mutation in low-risk groups (**Figure 9B**). KM curve was used to study the effect of TMB (**Supplementary Table 5**) on PRAD patients’ prognosis, results revealed that there was not significant BFFS difference between high and low TMB score groups which were classified depended on median TMB score (**Figure 9C**). However, we could find that this signature had great influence on patients’ prognostic no matter what TMB scores they had (p< 0.001, **Figure 9D**). Furthermore, the TMB was used to complete a correlation test between subgroups. PRAD patients in high-risk group exhibited increased TMB (p = 0.032, **Figure 9E**), indicating that they had more mutant tumor cells and that immunotherapy may be more effective for them.

### Analyze of tumor immune microenvironment and immunotherapy

**Figure 10:**
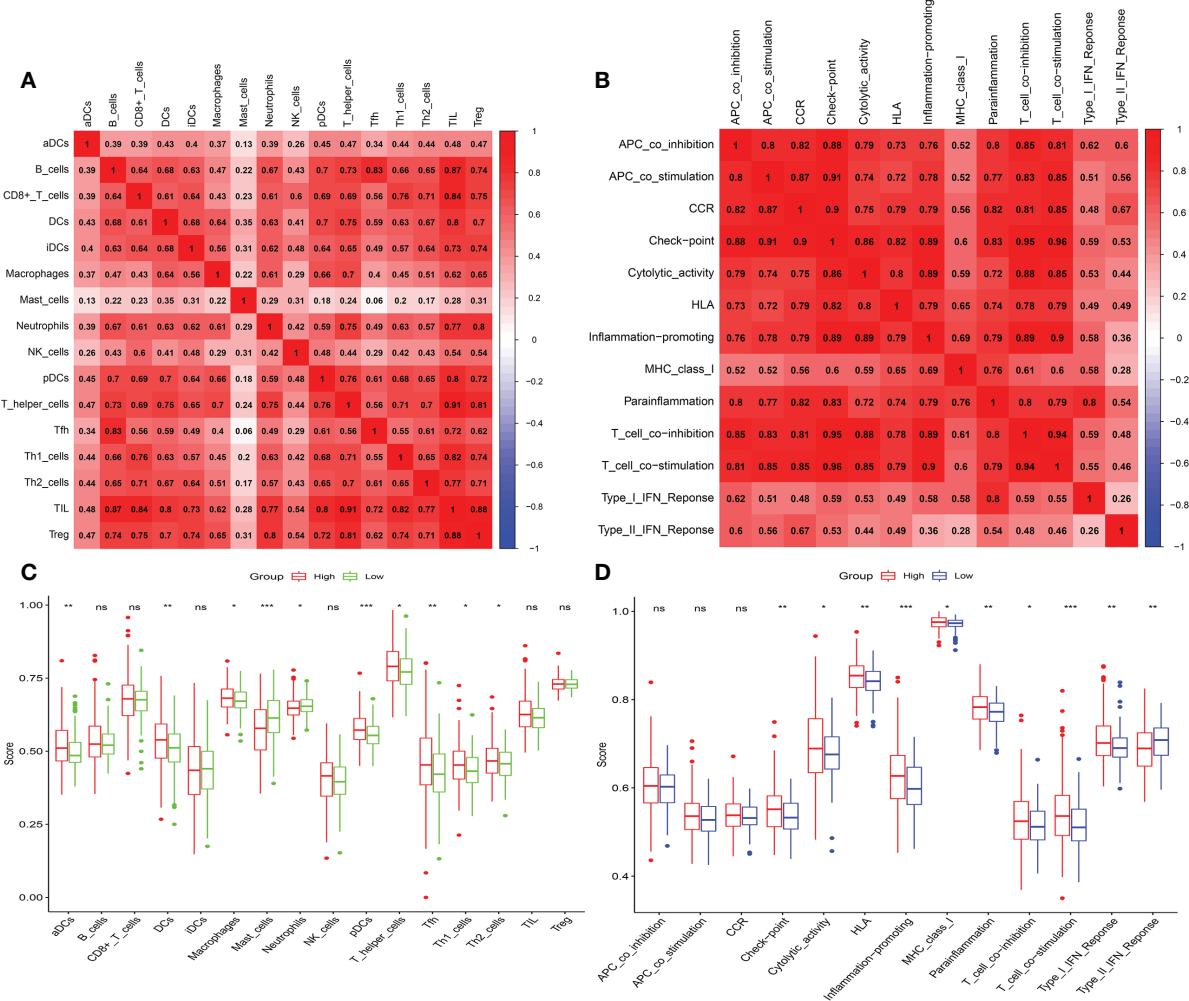
Immune infiltrating and correlation analysis. **(A)** Correlation between different immune cells. **(B)** Correlation between different immune-related pathways. **(C, D)** Analysis of enrichment scores of immune cells and immune-related pathways. (ns, p >= 0.05; *0.01 <= p < 0.05; **0.001 <= p < 0.01; ***p < 0.001).

**Figure 11:**
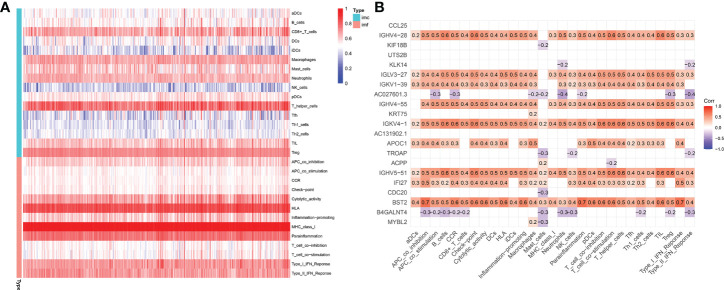
Immune microenvironment in TCGA-PRAD cohort. **(A)** Enrichment degree of immune cells and pathways in PRAD patients. **(B)** Association between prognostic-related DEGs and immune cells or pathways (DEGs: differential expression genes between high-risk and low-risk groups; red: positively correlated, violet: negatively correlated).

**Figure 12:**
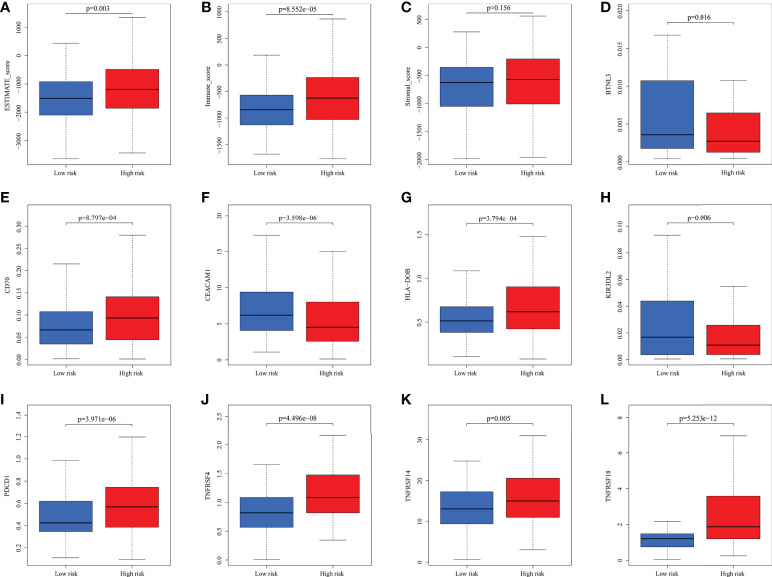
The analysis of immune scores and ICGs. **(A-C)** Pearson’s association analysis of ESTIMATE score, immune score and stromal score between subgroups. **(D-L)** Association analysis of some ICGs.

The microenvironment, which included immune cells, inflammatory factors, extracellular matrix, and different growths, had a significant influence on the diagnosis and treatment of cancer. To investigate the connection between various immune cells and immunological-related pathways, correlation analysis was performed on PRAD infiltrating immune subsets. **Figures 10A, B** represented the outcome of the association between immune subgroups that infiltrate tumors. There was a stronger association between immune cells or pathways if the correlation coefficient was closer to 1. SsGSEA was then used to examine enrichment scores of 16 immune cells and 13 pathways in TCGA, and 616 DEGs were discovered between subgroups. Result showed that high-risk group had significantly higher infiltration scores in immune cells (such as dendritic cell family, T follicular helper cells, and macrophages) and immune-related pathways (such as check-point, human leukocyte antigen, inflammation-promoting, T cell co-stimulation), while had lower scores in mast cells, neutrophils and Type II interferon (IFN) response (**Figures 10C, D** **)**. PRAD patients’ enrichment scores for these cells and pathways were visualized using a heatmap (**Figure 11A**). T helper cells, human leukocyte antigen and major histocompatibility complex (MHC) class I were found to be upregulated in PRAD. Among the 616 DEGs reported above, the univariate cox analysis discovered 21 prognostic-related DEGs with p< 0.001. **Figure 11B** demonstrated the enrichment of these genes in immune cells and pathways. We discovered a strong connection between *IGKV4-1*, *IGHV5-51*, and *BST* and these particular cells and pathways during our research. This signature was found to have a considerable impact on the immune microenvironment, which may benefit PRAD patients in terms of treatment and prognosis. To further investigate correlation between signature and immunity scores and immunotherapy, the correlation analysis of immune, estimate and stromal scores from ESTIMATE was performed between subgroups. Estimate score and immunological score were considerably higher in high-risk group (p< 0.05), indicating that this signature was highly related with the body’s immune system (**Figures 12A–C**). Then, differential expression analysis of ICGs between subgroups was employed by Wilcoxon-test with |log2FC | ≥ 0.4 and p< 0.05. The ICGs such as *CD70*, *HLA-DOB* and *PDCD1* were identified upregulated in high-risk group (**Figures 12D–L**). These ICGs had great potential to become new therapeutic targets, which may improve prognosis for PRAD patients.

### Correlation between risk score and sensitivity of drug to chemotherapy

**Figure 13:**
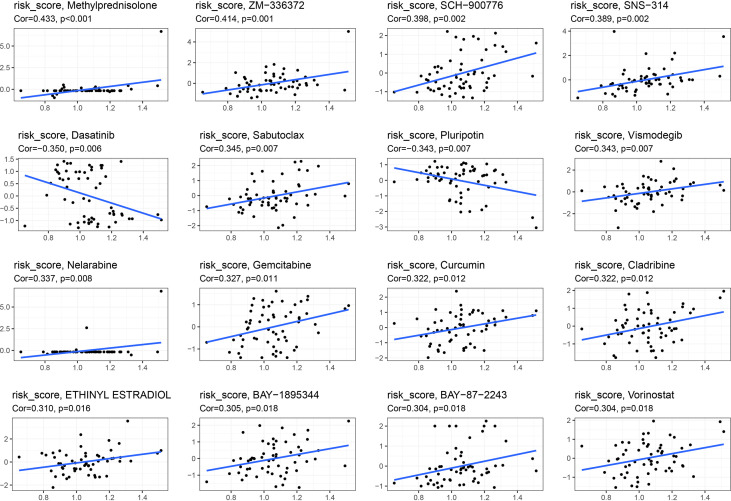
Association between this signature and drug sensitivity.

Pearson’s correlation analysis was used to examine correlation between the signature and drug sensitivity of NCI-60 cell lines. We found a positive correlation between this signature and methylprednisolone and sabutoclax, as well as a negative correlation between dasatinib and pluripotin and vismodegib (**Figure 13**).

## Discussion

In our study, we discovered a novel signature (*ARRB2*, *GARS*, *ODF2*, *PGK1*, *PKM*, *PLOD3*, *PNN*, *TYMP*, *WDR5*) based on moonlighting-related genes, which was significantly associated with prognostic, immune microenvironment and therapy of PRAD patients. This signature could be one independent prognostic factor to predict BFFS of PRAD patients. It was also associated with immune status and immunotherapy. So, our study suggested that the 9-gene signature deserves further study in clinical as a potential prognostic model.

In recent years, the prevalence of PRAD has been on the rise. Patients’ prognoses are still unpleasant, particularly for those with advanced tumors (3). Evidence suggests that the frequency of PRAD is influenced by the expression of certain genes, as well as environmental and lifestyle variables such pathogen infection, physical and chemical factors, and dietary factors (28). The significance of BCR genomic alterations in PRAD cannot be overstated, with ETS family mutations accounting for around 45% of PRAD patients (29). Nevertheless, a novel set of biomarkers to predict the occurrence, development, and outcomes of PRAD patients was required to be developed.

The idea of “moonlighting” proteins suggests that some proteins can have many functions, which lends credence to the idea that human monogenic diseases are phenotypically complicated (30). There are numerous moonlighting proteins (enzymes, transcription factors, scaffolds, adhesins, etc.) that offer molecular linkages between diverse biological processes. The moonlighting genes have several biological activities, including modulation of cell motility, angiogenesis, RNA splicing, and mRNA translation inhibition. Also mentioned is that the close association between moonlighting genes and cancer (31). 103 moonlighting genes for Homo sapiens were acquired. For example, one of the moonlighting genes, BRCA1, has been verified to associated with RNA polymerase II holoenzyme and acted as a transcription regulator (32). Our study analyzes the relationship between this signature and prognostic, immune microenvironment, TMB and drug sensitivity.

Among the 9 moonlighting genes from the signature, some have been reported in PRAD. *CXCR7*/*Src*/*EGFR*-mediated miyogenic signaling is negatively regulated by *ARRB2*, a tumor suppressor, which plays a crucial role in controlling *CXCR7*/*EGFR*-mediated tumor cell proliferation (33). For patients with *PRAD*, *ODF2*, an antigen identified by treatment-associated autoantibodies, could be considered as a feasible and customized immunotherapy option (34). Prostate cancer cells release *PGK1*, which regulates bone metastatic activity by increasing osteoblastic activity and decreasing osteoclastic function (35, 36). *PKM2*/*PKM* can be activated in PRAD patients to speed up transfer of extracellular vesicles to bone marrow stromal cells and control androgen responses to deal with the decreased androgen levels and hypoxia (37). Furthermore, *PKM2*/*PKM* play a crucial role in regulating cell cycle and tumor metabolism, including glycolysis (38). *PNN*, a desmosome-associated protein that regulates cell cycle, cell invasion, migration, and EMT processes, might be a viable therapeutic target for PRAD, and PNN can regulate PI3K/AKT and ERK/MAPK pathway (39), which contribute greatly to the development of PRAD (40). WDR5 has been reported to be a significant epigenomic integrator of histone phosphorylation and methylation, be an important driver of androgen-dependent PRAD cell proliferation and be upregulated in PRAD (41). Even though they have not been studied further in PRAD, the other genes have important biological functions as well. tRNA-charging enzyme *GARS* has been found as a biomarker for a variety of cancers. Apoptosis in hepatocellular carcinoma cells can be inhibited by reduction of *GARS*, whereas overexpression can accelerate cell growth, diminish xenograft necrosis, and increase *CD206+* tumor-associated macrophage infiltration (42). *PLOD3* has also been verified to be connected with colorectal cancer’s progression (43), lung cancer (44) and ovarian cancer (45). In triple-negative breast cancer, increased *TYMP* expression was significantly related with a positive reaction to capecitabine (46). Although *GARS*, *PLOD3* and *TYMP* have been studied extensively, the specific functions of the genes in PRAD have not been researched, which is deserved for further research.

A new predictive signature for PRAD depended on moonlighting genes had been validated in TCGA and GEO cohorts, which we analyzed in our analysis. Immune microenvironment and immunotherapy were also suggested to be associated to this signature. However, there are some limitations of this signature. When it comes to prognostic performance, for example, RNA-seq and clinical data were gathered from public sources, which may have some limitations. Artificial intelligence (AI) is helping researchers analyze larger data sets and provide faster, more accurate diagnoses of PRAD (47). AI combined with molecular characterization and radiogenomics could better enhance the accuracy of PRAD diagnosis and help choose the most appropriate treatment option, which can be further combined with our research (8). From this research, we selected 9 moonlighting genes as biomarkers to predict the progress and prognostic of PRAD, which may increase the costs for each patient, so our signature needs to be further improved in further research and simplify signature to construct a more accurate and effective signature. So, we need more experimental data and further research to assess prognostic value of this signature in PRAD. Further investigation is required on the specific mechanisms and functions of moonlighting genes to promote the progression of cancer (13).

In conclusion, our research uncovered a unique prognostic signature for PRAD depended on moonlighting molecular subtypes. There were not only significantly difference in their prognoses between high and low-risk groups, but also significantly difference in PRAD clinicopathologic characteristics, immune microenvironment, TMB and immunotherapy. To enhance the therapeutic impact and accomplish tailored therapy of PRAD based on the gene signature, more research will be conducted on subgroup-specific targeted treatment and biomarkers.

## Conclusion

In this work, we identified a unique prognostic signature associated with moonlighting, which could be an independent prognostic factor for PRAD patients. Besides, we discovered a strong relationship between this signature and the occurrence and development of PRAD, as well as the immunological microenvironment. It was closely correlated to immunological checkpoints and chemotherapeutic sensitivity.

## Data availability statement

Publicly available datasets were analyzed in this study. This data can be found here: The RNA-seq and clinical data could be acquired from TCGA (https://portal.gdc.cancer.gov/) and GEO database (https://www.ncbi.nlm.nih.gov/geo/). Data of immune, estimate and stromal scores of TCGA-PRAD samples were acquired from ESTIMATE database (https://bioinformatics.mdanderson.org/estimate). The data of drug sensitivity could be obtained from CellMiner (https://discover.nci.nih.gov/cellminer). Code used in our study are available from corresponding author on reasonable request.

## Ethics statement 

The studies involving human participants were reviewed and approved by Ethics Committee of Shanghai East Hospital affiliated to Tongji University. The patients/participants provided their written informed consent to participate in this study.

## Author contributions

WM is responsible for the research design, bioinformatic analysis and completing manuscript. LJ and BZ are responsible for statistical analysis. XS is responsible for the graph making and modification. GY, SL, and LY are responsible for providing technical guidance. LY provided the research funds. All authors approved the manuscript to be released and agreed to be responsible for all aspects of the work.

## Funding

This work was supported by grants from the National Natural Science Foundation of China (No.81972409, 81672549).

## Acknowledgments

Thanks to the TCGA, GEO ESTIMATE and CellMiner staff for the data.

## Conflict of interest

The authors declare that the research was conducted in the absence of any commercial or financial relationships that could be construed as a potential conflict of interest.

## Publisher’s note

All claims expressed in this article are solely those of the authors and do not necessarily represent those of their affiliated organizations, or those of the publisher, the editors and the reviewers. Any product that may be evaluated in this article, or claim that may be made by its manufacturer, is not guaranteed or endorsed by the publisher.

## Abbreviations

PRAD, prostate cancer; TCGA, The Cancer Genome Atlas; BFFS, biochemical failure-free survival; GEO, Gene Expression Omnibus; KM, Kaplan-Meier; ROC, receiver operating characteristic; AUC, areas under ROC curve; TMB, tumor mutation burden; PSA, Prostate-specific antigen; RP, radical prostatectomy; BCR, Biochemical recurrence; RNA-seq, RNA sequencing; mDEGs, m7G-related differentially expressed genes; ESTIMATE, Estimation of Stromal and Immune cells in Malignant Tumor tissues using Expression; ICGs, immunological checkpoint genes; LASSO, least absolute shrinkage and selection operator; HR, hazard ratio; CI, confidence interval; GSEA, Gene Set Enrichment Analysis; MSigDB , Molecular Signatures Database; GO, Gene Ontology; BP, Biological Process; CC, Cellular Component; MF, Molecular Function; KEGG, Kyoto Encyclopedia of Genes and Genomes; SNV, single nucleotide variation; GSVA, Gene Set Variation Analysis; PCA, Principal component analysis; ssGSEA, single-sample gene set enrichment analysis; NCI, US National Cancer Institute.
