# Supplementary material for: Computer classification and construction of a novel prognostic signature based on moonlighting genes in prostate cancer
Source: Front Oncol. 2022 Oct 7;12:982267. doi: 10.3389/fonc.2022.982267 (PMC9585316; doi:10.3389/fonc.2022.982267)
Supplement: Supplementary file 2 [file DataSheet_2.docx]

**Supplementary Table1**

The 103 Homo sapiens moonlighting genes’ list and functions.

**Supplementary Table2**

The immune score, estimate score, and stromal score of TCGA-PRAD samples obtained from ESTIMATE database.

**Supplementary Table3**

The list of immunological checkpoint genes.

**Supplementary Table4**

The results of differential expression analysis of moonlighting genes.

**Supplementary Table5**

The tumor mutated burden scores of prostate cancer patients in TCGA.
